# Near-Infrared Spectroscopy Is Promising to Detect Iliac Artery Flow Limitations in Athletes: A Pilot Study

**DOI:** 10.1155/2018/8965858

**Published:** 2018-12-20

**Authors:** Martijn van Hooff, Goof Schep, Eduard Meijer, Mart Bender, Hans Savelberg

**Affiliations:** ^1^Department of Sports and Exercise, Máxima Medical Centre, De Run 4600, 5500 MB Veldhoven, Noord-Brabant, Netherlands; ^2^Department of Human Movement Science, Faculty of Health Science, Maastricht University Maastricht, Postbus/P.O. Box 616, 6200 MD, Maastricht, Limburg, Netherlands; ^3^Department of Clinical Physics, Máxima Medical Centre, De Run 4600, 5500 MB Veldhoven, Noord-Brabant, Netherlands; ^4^Surgery Department, Máxima Medical Centre, De Run 4600, 5500 MB Veldhoven, Noord-Brabant, Netherlands

## Abstract

Endurance cyclists have a substantial risk to develop flow limitations in the iliac arteries during their career. These flow limitations are due to extreme hemodynamic stress which may result in functional arterial kinking and/or intravascular lesions. Early diagnosis may improve outcome and could prevent the necessity for surgical vascular repair. However, current diagnostic techniques have unsatisfactory sensitivity and cannot be applied during exercise. Near-infrared spectroscopy (NIRS) has shown great diagnostic potential in peripheral vascular disease and might bring a solution since it measures tissue oxygenation in real time during and after exercise. This report describes the first experiences of the application of NIRS in the vastus lateralis muscle during and after maximal graded cycling exercise in ten healthy participants and in three patients with flow limitations due to (1) subtle functional kinking, (2) an intravascular lesion, and (3) severe functional kinking. The results are put into perspective based on an empirically fitted model. Delayed recovery, showing clearly different types of patterns of tissue reoxygenation after exercise, was found in the affected athletes compared with the healthy participants. In the patients that had kinking of the arteries, tissue reoxygenation was clearly more delayed if NIRS was measured in provocative position with flexed hip. In this pilot experiment, clearly distinctive reoxygenation patterns are observed during recovery consistent with severity of flow limitation, indicating that NIRS is a promising diagnostic tool to detect and grade arterial flow limitations in athletes. Our findings may guide research and optimization of NIRS for future clinical application.

## 1. Introduction

Arterial flow limitations in young otherwise healthy adults are unusual. In contrast, approximately one in five professional cyclists will eventually develop a sport-related flow limitation in the iliac arteries [1]. The prevalence in recreational cyclists is unknown. The typical complaints of this condition are pain and loss of power at near-maximal exercise, which rapidly disappear during recovery.

In athletes, the flow limitation may be caused by intravascular lesions, functional kinking of the vessels, or a combination of both [1–4]. Compared to nonathletes with intravascular lesions that are mainly atherosclerotic, the intravascular lesions in athletes are caused by endofibrosis, a histologically different entity [5].

Schep* et al.* described that repetitive kinking can lead to flow limitations per se, which can be objectified by ultrasound and Magnetic Resonance Angiography (MRA) in complaint provoking positions [6, 7]. Surgical treatment for kinking, encompassing release of the iliac arteries, proved to be effective [1, 4]. Despite the literature on the existence of functional kinking, it remains a subject of debate whether kinking can result in flow limitations in the absence of intravascular lesions [6–8]. Flow limitation due to kinking seems to be an early stage of the disease and these early stages are difficult to diagnose, which may contribute to the current controversy [5, 6, 9]. There is agreement among experts that kinking may result in an increase in the hemodynamic load in the vessel with substantially increased peak systolic velocities, which may lead to intravascular damage and a resultant endofibrotic reaction [5, 6].

In the more advanced stages of the disease, with substantial intravascular lesions, diagnostic accuracy of standard tests is high. It was observed that the Ankle Brachial Index (ABI) measured within five minutes after ceasing a maximal exercise test on a recumbent bike has a sensitivity of 90% and a specificity up to 87% [10]. However, in the earlier stages of the disease with predominantly kinking as an underlying cause and without significant intravascular lesion, diagnosis is a major challenge. The best single test is the maximal, provocative exercise test on a cycle ergometer followed by measuring ABI in a competitive posture. In the rare case that the problem is unilateral, the sensitivity is 73%. If the problem is bilateral, the sensitivity is only 43% [3].

In advanced stages, vascular reconstructive surgery with the use of venous patch may be indicated. If the disease is detected at an earlier stage, less invasive surgical treatment, consisting of release of the iliac artery, is possible and it might also be possible to prevent progression of the disease by adaptations in sports [1, 4, 11, 12]. Therefore, it is of substantial clinical importance to develop new, more sensitive measurement techniques to detect these subtle flow limitations. In particular, techniques that measure the direct (patho)physiological effect of a flow limitation during exercise might be promising. Near-infrared spectroscopy (NIRS) provides an optical estimate of (muscle) tissue oxy- and deoxyhemoglobin concentration. As the instrument is small and the measurements are noninvasive and in real time, it can be used during and after exercise [13, 14].

NIRS has been used to determine the presence and severity of peripheral atherosclerotic vascular disease (PAD) [15–17]. To our knowledge, no literature exists on the use of NIRS in athletes with iliac artery flow limitations. The goal of this study is to explore whether NIRS can be applied during a clinical exercise test in athletes with suspected flow limitations in the iliac arteries, and whether cycling posture, upright, and competitive posture, respectively, might add extra diagnostic information regarding subtle flow limitations associated with arterial kinking. This report gives the first experiences of NIRS in ten healthy reference subjects and three typical patients with proven flow limitations and presents a pathophysiological model to explain the observed results.

## 2. Methods

### 2.1. Subjects

Three patients from the vascular clinic of Máxima Medical Centre, Veldhoven, Netherlands, were recruited. The patients represented typical examples of persons suffering from sport-related flow limitations due to, respectively, subtle functional kinking, an intravascular lesion, and severe functional kinking. As a reference, a well-trained, healthy athletic male (cycling more than 15 cycling hours per week and competing on a national competitive level) without complaints suspect for arterial flow limitations was recruited. In addition, nine healthy male athletes in the same age range as the patients (29.6 s.d +/- 2.2 years) were included to demonstrate the variation in NIRS values. These subjects were recruited from ongoing research on cycling in competitive posture (not yet published). All subjects were nonsmokers. All subjects gave written informed consent. The study was approved by the Medical Ethical Review board of the Máxima Medical Centre, Veldhoven. The study was conducted in accordance with the declaration of Helsinki. All subjects provided written informed consent.

### 2.2. Echo-Doppler and Magnetic Resonance Angiography

All subjects underwent an Echo-Doppler examination (Terason T3000, Burlington, MA, USA) with colour coding facility, performed by an experienced vascular technician. This examination is able to visualize the iliac arteries on vascular abnormalities and scale the severity by measurements of the peak systolic velocity (PSV). In addition, patients were screened using a 1.5 Tesla MRA (Achieva, Philips, release 3.2.3.2., Best, Netherlands). These examinations were performed with an extended hip joint to visualize intravascular abnormalities and with a flexed hip joint to demonstrate kinking [6, 7, 9].

### 2.3. Cycling Test and Cardiopulmonary Measurements

**Figure 1:**
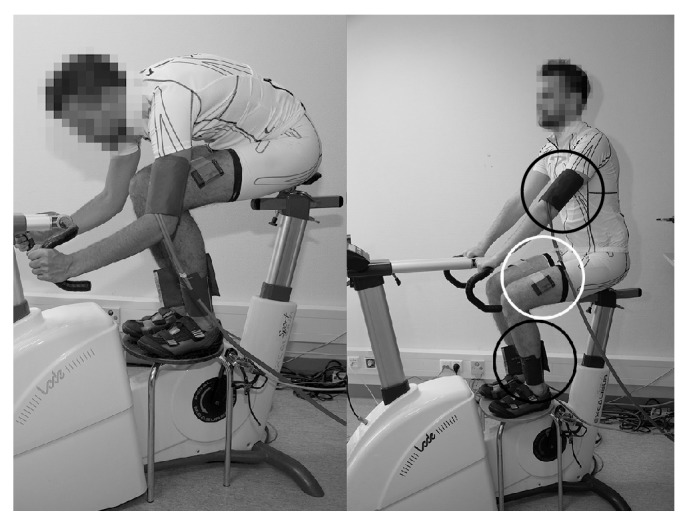
Visual presentation of the used postures (CP and NP for the left and right picture, resp.). This was also the racing position during cycling. In the photographs, the position in recovery is illustrated when measuring ABI (black circles denotes the placement of the blood pressure cuffs while the white circle denotes the NIRS devices without the black cloth in order to expose the measurement device in this picture).

The healthy reference subject and the patients performed on an electromagnetically braked cycle-ergometer (Excalibur Sport, Lode, Groningen, Netherlands) two provocative, maximal, cardiopulmonary exercise tests with breath-by-breath measurements of gas exchange and ventilatory parameters (Quark CPET, Cosmed, Rome, Italy, or Vyntus CPX, CareFusion, Höchberg, Germany). The first exercise test was in competitive posture (CP) with the trunk almost in a horizontal position to provoke possible kinking and complaints as much as possible; the second test was in a normal posture (NP) with the trunk in a vertical position (Figure 1). During both tests, the subjects were instructed to keep a preferred, constant pedalling frequency between 80 and 100 rotations per minute. The protocol consisted of four minutes pedalling at 10% of the maximal workload, estimated based on gender, weight, and physical fitness as a warm-up. This was followed by an individualized, maximal ramp exercise test aimed at reaching exhaustion within 8-12 minutes [18]. The additional nine healthy subjects performed this test only in CP to demonstrate the variation in NIRS values. The peak workload was defined as the last registered workload. The VO_2max_ (ml/min) was recorded as the highest 15-breath average value prior to volitional exhaustion. VO_2max_ was normalized for body mass to allow comparison between subjects and to grade exercise capacity. After maximal exhaustion, subjects were instructed to detach from their fixed shoe pedal connection as fast as possible and position their feet on a resting platform which was placed at the area of the bike pedals over the bike. During the recovery phase, the subjects had to remain seated in CP in order to provoke and maintain kinking of the iliac artery if existent or in NP, respectively. Immediately following positioning of the resting platform, five subsequent simultaneous blood pressure measurements at the arm (Critikon 1846-SX, Soma Technology, Highland park Dr., Bloomfield, USA) and ankles (Duo, Datascope Corp., Mahwah, USA) in order to calculate the Ankle Brachial Index (ABI) [2]. The ABI was calculated as:

(1) $$\begin{array}{c} ABI=\frac{SAP-\left(D_{ab}*0.76\right)}{SBP} \end{array}$$

where SAP is the systolic ankle pressure in mmHg, SBP is the systolic brachial pressure in mmHg and D_ab_ is the vertical height difference correction between the ankle and arm in cm (1 cm = 0.76). Each ABI reading was possible after approximately 1 minute. Diagnostic thresholds for the ABI in detecting flow limitations in completive cyclists in this position were 23 mmHg pressure difference between the ankles or an ABI of <0.54 [2, 3].

### 2.4. NIRS Measurements

To measure changes in oxygenated haemoglobin (O_2_Hb) and deoxygenated haemoglobin (HHb) in muscle tissue, a 10 Hz PortaMon NIRS device (PortaMon, Artinis, Elst, Netherlands) was used. The device uses the modified Lambert Beer law with spatially resolved spectroscopy and two wavelengths of emitting light (760 and 850 nm) with three spatially configured (30, 35, and 40mm) light emitting diodes and a photo detector diode. A differential path length factor of four was used, as recommended by the manufacturer.

An absolute measure of the oxygen saturation index (TSI, equalling (O_2_Hb/(O_2_Hb + HHb) x 100)) can be calculated from the coefficients of absorption which are derived from the light attenuation slopes at different wavelengths and source-detector distances using the photon diffusion theory [19].

TSI and O_2_Hb were used to operationalize abnormal supply of arterial blood flow. HHb gives information on the O_2_ extraction and outflow of blood which is not restricted in this entity. On each leg, a NIRS device was placed 15 centimetres proximal from the proximal patella edge on the muscle belly of the vastus lateralis muscle with adhesive tape and a black cloth to eliminate the influence of ambient light on the measurements. The penetration depth of near-infrared light is approximately half the distance between the source and the detector. To minimize confounding of cutaneous and subcutaneous layers, subjects with an adipose tissue thickness of more than 7.5 mm (15 mm skinfold thickness) were excluded [13, 20, 21]. ATT was estimated as half of the skinfold thickness at the site of the NIRS measurement (Harpenden; Baty International, West Sussex, UK). All NIRS measurement data were saved for further analysis.

### 2.5. Data Analysis

**Figure 2:**
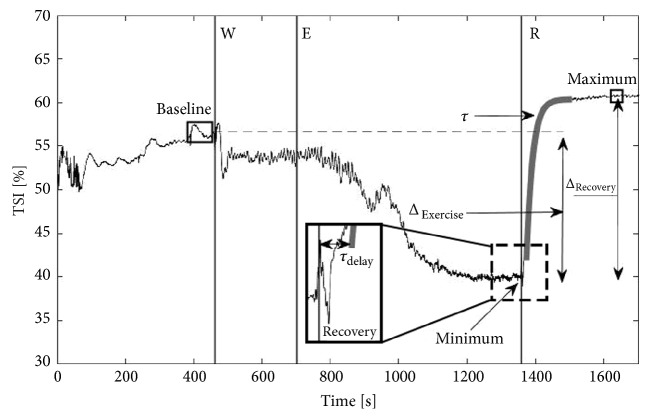
Visual presentation of TSI with the variables during and after the maximal provocative exercise. W: warm-up; E: exercise; R: recovery. The thick grey curve represents the best fit of the monoexponential model (tau).

First, outliers (e.g., unwanted movements of the body) in TSI and O_2_Hb data were detected using a Hampel filter that replaces the value with the median if exceeding three standard deviations from the median of itself and three neighbouring data points of that median value [22]. Thereafter, all TSI and O_2_Hb data were filtered with a 10^th^ order Butterworth low pass filter with a cut-off frequency of 1 Hz. To characterize TSI and O_2_Hb patterns, baseline, minimal, and maximal values and ranges were calculated. TSI_minimum_ and O_2_Hb_minimum_ were calculated as the 5-second bandwidth around the minimal value (average of the point itself and the 50 neighbouring data points) after ceasing the exercise. Similarly, TSI_maximum_ and O_2_Hb_maximum_ were calculated as the 5-second bandwidth around the maximally attained value during the recovery phase (average of the point itself and the 50 neighbouring data points) (Figure 2). While NIRS is able to measure concentration changes, the O_2_Hb signal was set to an arbitrary zero baseline at the start of the warm-up phase (O_2_Hb_baseline_) in order to measure time-dependent concentration change relative to the start of the warm-up phase. The amplitude of the exercise (TSI_Δexercise_) was calculated as the difference between the 10-second average prior to the start of the warm-up phase (TSI_baseline_) and the TSI_minimum_. The amplitude of the recovery (TSI_Δrecovery_; O_2_Hb_Δrecovery_) was calculated as the difference between the minimum (TSI_minimum_; O_2_Hb_minimum_) and the maximal (TSI_maximum_; O_2_Hb_maximum_) value.

### 2.6. Kinetic Analysis

Due to the lack of a physiology-based model in our patient population, an empirical monoexponential model was used using the least squares method to be able to describe and quantify the data. This approach is well known from literature [23–26]. This was done in a custom-made analysis program (MatLab 2013a [8.1.0.604], Mathworks, Natick, USA) with the following equation:

(2) $$\begin{array}{c} Y\left(\;t\;\right)=Y_{minimum}+Y_{\Delta recovery}*\left(\;1-e^{\left(t-Td\right)/\tau }\;\right) \end{array}$$

where Y(t) stands for the type of signal, TSI, or O_2_Hb, respectively, of which Y_minimum_ is the minimum value, Y_Δrecovery_ is the recovery value, t is the time, T_d_ is the time delay which is defined as the time between the start of the recovery phase and the start of the fit (Figure 2), and *τ* is the empirical time constant associated with the recovery. The recovery phase was visually marked if no cycling-induced cyclic pattern was observed in the NIRS signals. The start of the monoexponential function was determined by the highest cross-correlation by fitting a monoexponential shaped function over the signal [27]. The value that was not exceeded by another higher value within 30 seconds was considered the end-point of the fit. Lastly, to take the delay into account which is expected to be more prominently present in patients during the test in CP, the mean response time (MRT) was calculated by the sum of the time constant and the time delay. The goodness of fit was defined by the coefficient of determination (R^2^). In addition, the half-value time (HVT) was calculated which is defined as the time taken to reach half the value between the minimal and the maximal value from the recovery phase.

## 3. Results

### 3.1. Artery Visualisation

**Figure 3:**
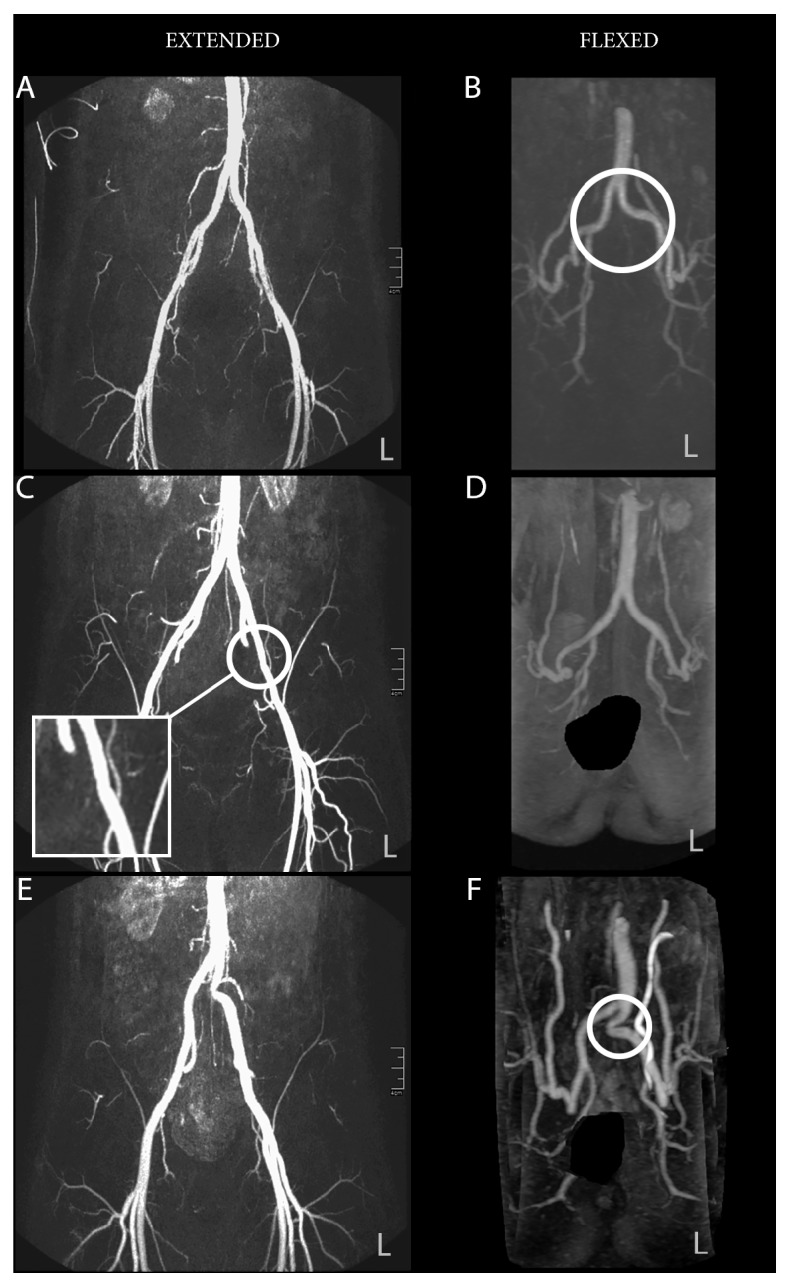
The first patient (A, B) shows subtle kinking, the second patient (C, D) shows an intravascular lesion, and the third patient (E, F) shows severe kinking. The circles denote the place of abnormality in the artery.

The healthy subjects showed normal findings with Echo-Doppler examination. The first patient was a competitive cyclist with complaints due to a sport-related flow limitation in his left leg. The Echo-Doppler showed minimal lengthening in the left common and external iliac artery with subtle functional kinking in the flexed hip position. This was halfway the common iliac artery and at the iliac bifurcation; this kinking aggravated after exercise. This led to two kinking angles slightly less than 90 degrees and increased PSV (flexed hip >1.76 m/sec and psoas contraction >1.70 m/sec) on the left side [6]. The right side also showed subtle kinking of the vessel in the external iliac artery in rest but this stretched out during exercise. No intravascular lesions were found. The MRA (Figure 3(a)) with extended hips showed minimal lengthening in the common and external iliac artery with a minor intravascular lesion on the left side. The MRA (Figure 3(b)) with flexed hip showed subtle bending of the vessels at the same places as kinking was observed with echo. The patient had no complaints on his right leg despite minimal lengthening and a minor intravascular lesion. The second patient was a competitive cyclist with complaints due to a sport-related flow limitation in his left leg. Echo-doppler showed an intravascular lesion proximal in the left external iliac artery. Additionally, the vessel showed deep bending of the vessel inducing a possible minor kink. Despite the absence of complaints on his right leg, an intravascular narrowing was observed over the entire external iliac artery. This arterial abnormality was in a lesser extent than the left leg. Both legs showed abnormal PSV whereas the left side showed the highest values. The MRA (Figure 3(c)) showed in the area of intravascular narrowing a diameter of the external iliac artery of 65% (left side) and 75% (right side) compared to the distal healthy reference part of the narrowing vessel. No functional kinking was observed (Figure 3(d)). The last patient was a competitive cyclist with complaints on the left side. Echo-Doppler showed severe functional kinking in the common iliac artery with some intravascular lesion. The MRA (Figures 3(e) and 3(f)) shows excessive length and severe kinking of the left common iliac artery with dilatation of the distal common iliac artery [7, 9]. On the right side, only minor lengthening of the common iliac artery and external iliac artery without intravascular abnormalities was seen.

### 3.2. Ankle Brachial Index

In the healthy subjects, ABI values were normal (Table 1). In the person with subtle kinking (patient 1), ABI values were normal in NP but were abnormal at the left side in CP, and the right-side ABI was normal. In the participant with an intravascular lesion (patient 2), ABI values on the left side were abnormal in both NP and CP, while no abnormal values were found on the right side. In the subject with severe kinking (patient 3), in CP the left ankle pressure was too low to measure for four minutes, while in NP it was within the normal values.

### 3.3. NIRS Results

**Figure 4:**
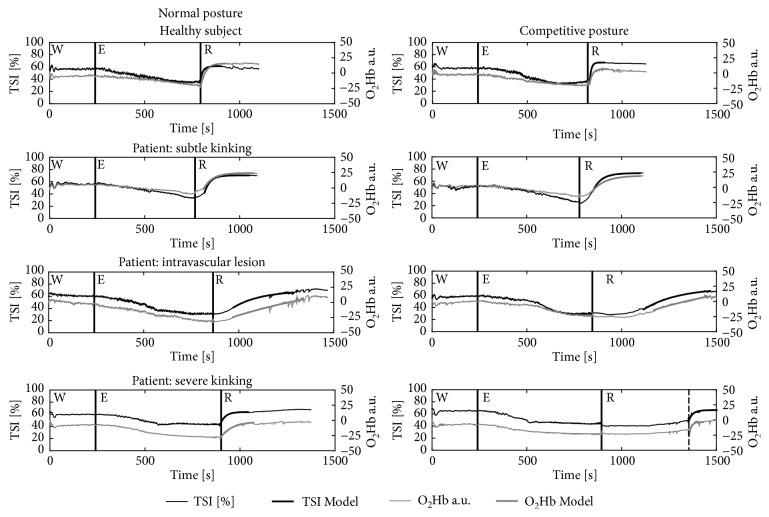
TSI and O_2_Hb patterns during warm-up (W), RAMP exercise protocol (E), and recovery (R); solid vertical lines denote phase transitions, the dashed line in CP with severe kinking represents the sudden change from CP to NP.

**Figure 5:**
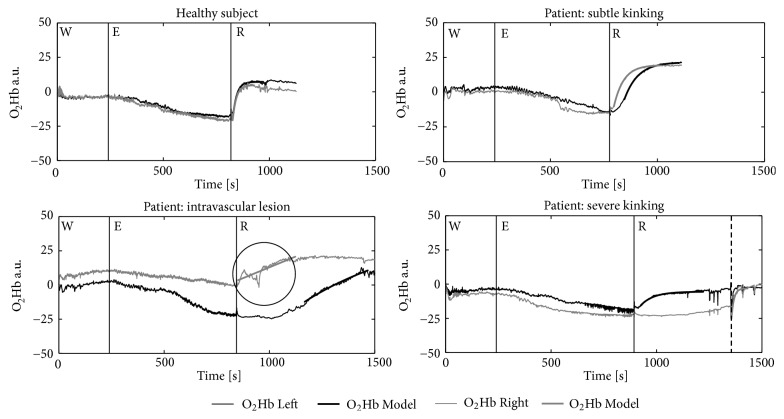
O_2_Hb patterns of the left (black) and right (red) leg in CP during warm-up (W), RAMP exercise protocol (E), and recovery (R); solid vertical lines denote phase transitions. The dashed line in CP with severe kinking represents the sudden change from CP to NP to demonstrate the effect of severe kinking on oxygenation. The data within the black solid circle in the patient with an intravascular lesion shows inaccurate data due to movement artefacts of the patients' right leg which slipped of the resting platform.

During the test but in particular during recovery, hardly any outliers were identified. The minimal outliers detected were most certainly due to unwanted body movements. During and after the cycling test, the absolute values and amplitude changes of O_2_Hb and TSI showed no clear differences between or within the four participants. However, remarkable differences occurred in the recovery kinetic variables (Table 2 and Figure 4); i.e., in the healthy subject, the rate of reoxygenation was substantially faster in each leg than in the patients, in both NP and CP (Figure 4). Between the healthy subjects, no clear differences were found between the legs or postures (Figure 5). The additional 9 healthy athletes in CP showed approximately similar NIRS results as the healthy reference subject (Table 2). In the three patients, the delayed reoxygenation was more prominent in CP than in NP. Moreover, the most affected leg showed the greatest delayed reoxygenation. In the patient with severe kinking, even no reoxygenation response was seen in CP. The sudden increase seen in Figure 4 after approximately eight minutes (dashed line) is due to sudden extending into NP which was accompanied by a gradual disappearance of the leg complaint of the athlete.

## 4. Discussion

Detecting and scaling the severity of (subtle) flow limitations in the iliac arteries in athletes are a major challenge and current techniques fail, especially in the early stages. NIRS noninvasively measures tissue oxygenation and relative change in oxygenated haemoglobin during and after exercise. The clear differences in NIRS variables, in line with the severity of flow limitations of participants, indicate that NIRS can be a promising addition in the diagnosis of flow limitations in the iliac arteries.

No clear differences in the healthy participants and participants with varying degrees of complaints were found in baseline tissue oxygenation and the pattern of tissue oxygenation during exercise (Table 2). In contrast, we observed striking differences in tissue oxygenation patterns following exercise between the healthy athletes and athletes with flow limitations. This is in line with studies in patients suffering from PAD after exercise or occlusion [16, 28–30]. The delay until the start of the monoexponential rise seemed to be related to the severity of the flow limitation as demonstrated by imaging. Also, our data suggested that abnormalities in oxygenation patterns after exercise increased due to provocative kinking of the vessel.

The flow through the artery is related to the perfusion of the muscle and inversely to the vascular resistance. Poiseuille's law shows the enormous influence of the vessel diameter on the rate of blood flow rate (*Q* = (ΔPr^4^*π*/*ηL*8)) [31], where Q is the flow rate (cm^3^/sec), P is the pressure (mmHg), r is the radius of the vessel (m), *η* is the viscosity of blood (Pa*∗*s), and L is the length of the vessel (cm). This law shows that the radius and thus cross-sectional area calculated as *π*r^2^ have a high influence on the flow rate distal to the intravascular lesion or kinking of the vessel. Adaptations of the radius changes the flow rate to the fourth power. Thus, reduction in radius may result in decreased limb systolic pressure as seen in the ABI [32].

Major differences in oxygenation recovery patterns were found in the patient with diagnosed severe functional kinking performing the test in NP and in CP. Hardly any recovery response was observed in the CP position (Figure 4). As described earlier, decreased cross-sectional area will result in a drop of flow rate and a decreased systolic pressure after the narrowing. If the intramuscular pressure increases due to exercise exceeding the arterial pressure, the blood flow may halt completely, which may explain the pattern observed in this patient [33]. In an upright posture, a substantial improvement is observed in the kinetic variables, which is likely to be caused by the increased arterial pressure now exceeding the intramuscular pressure.

The finding that in both patients (patients 1 and 3) with kinking there is substantial slower oxygenation recovery patterns in the CP versus the NP gives additional proof that functional kinking might be the major cause of the flow limitation in these athletes.

Previous research also showed correlation between reoxygenation and ABI in patients with PAD [29, 30]. NIRS estimates perfusion and oxygenation directly in working muscle and, therefore, might be a more sensitive diagnostic tool of flow limitation than ABI. The oxygenation patterns, as shown in Figure 4 and Table 2, seem promising when it comes to early stage detection of flow limitation. The differences that we observed in NIRS response from healthy subjects to severe flow limitation are large. In legs with minimal flow limitation in imaging when ABI was still in the normal range, NIRS values were abnormal.

This report represents a pilot which indicates that NIRS might give useful diagnostic information to detect and scale the severity of sport-related vascular problems. However, before NIRS can be introduced as a standard technique in the clinic, several steps have to be taken. Firstly, the test-retest reliability of the variables obtained with NIRS needs to be determined. Secondly, further studies are needed to ascertain what the best discriminating and robust diagnostic variables are from the oxygenation patterns that can be obtained from the NIRS measurement. Comparison studies between patients with pathology and controls need to be performed to determine the added clinical value of NIRS in relation to the other current diagnostic techniques. Also, in order to further demonstrate and scale kinking as a cause for flow limitation, a study comparing the oxygenation patterns in CP and NP in patients and healthy subjects may give a better insight in the clinical meaning of kinking in the occurrence of flow limitation and this may help to tailor treatment.

## 5. Conclusion

Our results indicate that NIRS is a promising diagnostic tool to detect subtle flow limitations in athletes during exercise. The rate of recovery in the recovery phase after a maximal exercise test seems to have a diagnostic value. This report encompasses only a small group of patients and one healthy subject. Therefore, further research is necessary to clearly validate NIRS its diagnostic power and to optimize its accuracy.

## 6. Perspective Paragraph

Approximately one in five professional cyclists will eventually develop a sports-related flow limitation in the iliac arteries. With current exercise testing up to 25% (one-sided problem) or 50% (two-sided problem) are not detected. NIRS may add important diagnostic sensitivity.

## Figures and Tables

**Table 1:** Exercise information in NP and CP. † values of 9 healthy athletes measured in CP; ^∗^Abnormal values for ABI are <0.54 and a pressure difference of >23 mm Hg or <-23 mm Hg; ^∗∗^First measurement possible after four minutes which was 0.54 [3].

	**Healthy competitive cyclist(s)**			**Patient: subtle kinking**		**Patient: Intravascular lesion**		**Patient: Severe kinking**	
Age	**19**		**29.6 (2.2)** ^†^	**38**		**37**		**27**	
Height (cm)	**190.6**		**183.7 (4.4)** ^†^	**187.1**		**175.4**		**180.2**	
Weight (kg)	**69.9**		**73.9 (4.4)** ^†^	**85.5**		**68**		**71.7**	
VO_2max_/kg (ml/min/kg)	**72.1**		**65.3 (10.1)** ^†^	**53.9**		**62.6**		**64.4**	

ATT Left (mm)	**3.6**		**3.13 (0.97)** ^†^	**2.9**		**2.5**		**3.6**	
ATT Right (mm)	**3.6**		**3.11 (0.94)** ^†^	**2.5**		**3.8**		**4**	

**Cycling test**									
	**NP**	**CP**	**CP**	**NP**	**CP**	**NP**	**CP**	**NP**	**CP**

Workload (Watt)	489	510	446.2 (42.8)^†^	420	427	381	370	457	432
Arm systolic pressure (mm Hg)	159	223	202.6 (16.5)^†^	117	161	165	174	108	144
Ankle systolic pressure left (mm Hg)	222	165	195.1 (23.3)^†^	168	114	120	127	155	0^∗∗^
Ankle systolic pressure right (mm Hg)	245	180	188.6 (26.2)^†^	184	147	182	182	185	136
Ankle systolic pressure difference (mm Hg) with respect to the right ankle	-23	-15	6.6 (12.6)^†^	-16	-33^∗^	-62^∗^	-55^∗^	-30^∗^	-136^∗∗^
ABI Left	1.00	0.58	.75 (0.09)^†^	0.72	0.47^∗^	0.43^∗^	0.53^∗^	0.66	0^∗∗^
ABI Right	1.15	0.64	.72 (0.11)^†^	0.86	0.68	0.80	0.85	0.94	0.69

**Table 2:** Absolute TSI values are expressed in % and amplitude changes of O2Hb in *μ*M. Kinetic values are expressed in seconds; *τ*, time constant of the monoexponential model; delay, Time delay; MRT, mean response time; HVT: Half value time; † represents average values with s.d. from 9 healthy athletes with, respectively, 18 legs in CP; ^∗^Most affected leg; ^∗∗^Patient leg slipped of the resting platform resulting in inaccurate data; ^∗∗∗^No (kinetic) recovery response present.

	Absolute (TSI) and amplitude changes (O_2_Hb)																
	Healthy top cyclist					Patient: Subtle kinking				Patient: Intravascular lesion				Patient: severe kinking			
	NP		CP			NP		CP		NP		CP		NP		CP	
	Left	Right	Left	Right	n=18^†^	Left^∗^	Right	Left^∗^	Right	Left^∗^	Right	Left^∗^	Right	Left^∗^	Right	Left^∗^	Right

TSI_baseline_	55.7	56.5	59.7	58.3	61.1 (3.8)^†^	56.1	55.2	50.4	53.5	61.4	62.7	57.6	60.6	59.8	63.7	64.8	63.3
TSI_minimum_	40.6	36.7	40.9	37.2	42.3 (8.3)^†^	34.2	31.9	26.6	29.6	30.8	47.1	29.9	46.6	43.7	47.6	44.0	46.1
TSI_maximum_	68.6	62.3	66.9	67.4	68.7 (2.7)^†^	70.8	67.3	73.5	71.4	72.2	69.4	67.6	*∗∗*	68.0	67.9	*∗∗∗*	67.9
TSI_∆exercise_	15.1	19.8	18.8	21.1	26.3 (8.1)^†^	21.9	23.3	23.8	23.9	30.6	15.6	27.7	14	16.1	16.1	20.8	17.2
TSI_∆recovery_	28	25.6	26	30.2	23.8 (13.0)^†^	36.6	35.4	46.9	41.8	41.4	22.3	37.7	*∗∗*	24.3	20.3	*∗∗∗*	21.8

O_2_Hb_minimum_	-15.7	-21.9	-18.5	-19.6	-18.7 (6.7)^†^	-10.6	-15.5	-14.0	-15.1	-31.8	-28.7	-23.2	-7.6	-27.0	-24.9	-22.4	-19.6
O_2_Hb_maximum_	20.2	15.3	6.9	5.6	9.2 (7.0)^†^	25.0	21.4	18.2	21.0	4.9	-4.9	10.3	*∗∗*	-2.2	0.9	*∗∗∗*	-7.7
O_2_Hb_∆recovery_	35.8	37.2	25.3	25.3	27.7 (9.4)^†^	35.6	36.9	32.2	36.1	36.7	23.8	33.5	*∗∗*	24.8	25.8	*∗∗∗*	12.0

	Kinetic values																
	Healthy top cyclist					Patient: Subtle kinking				Patient: Intravascular lesion				Patient: severe kinking			
	NP		CP			NP		CP		NP		CP		NP		CP	
	Left	Right	Left	Right	n=18^†^	Left^∗^	Right	Left^∗^	Right	Left^∗^	Right	Left^∗^	Right	Left^∗^	Right	Left^∗^	Right

TSI_*τ*_	14.7	14.9	11.4	9.2	15 (4.0)^†^	30.2	30.8	44.0	27.5	150.1	18.0	183.2	*∗∗*	26.4	28.3	*∗∗∗*	78.7
TSI_delay_	-1.2	-3.7	12.1	10.3	11 (8.1)^†^	50.1	41.8	69.8	19.7	106.6	13	269.9	*∗∗*	-7	-4.6	*∗∗∗*	32
TSI_MRT_	13.5	11.2	23.5	19.5	26 (11.2)^†^	80.3	72.6	113.8	47.2	256.7	31.0	453.1	*∗∗*	19.4	23.7	*∗∗∗*	110.7
TSI_R_^2^	0.980	0.975	0.994	0.987	0.989 (0.010)^†^	0.997	0.996	0.997	0.992	0.980	0.997	0.995	*∗∗*	0.987	0.993	*∗∗∗*	0.921
TSI_HVT_	11.6	7.3	20.1	18.0	21 (8.4)^†^	65.0	49.5	82.2	36.5	173.9	24.8	345.3	*∗∗*	22.2	18.3	*∗∗∗*	91.8

O_2_Hb_*τ*_	25.9	26.2	18.6	15.1	20.5 (3.8)^†^	43.9	37.5	66.9	43.3	747.0	31.3	554.2	*∗∗*	60.3	28.5	*∗∗∗*	64.8
O_2_Hb_delay_	-1.2	-1.7	11.0	9.8	11 (6.9)^†^	47.6	41.9	68.5	19.3	105.0	10.2	323.8	*∗∗*	-4.6	-1.6	*∗∗∗*	31.2
O_2_Hb_MRT_	24.7	24.5	29.6	24.9	31.5 (8.6)^†^	91.5	79.4	135.4	62.6	852.0	41.5	878.0	*∗∗*	55.7	26.9	*∗∗∗*	96.0
O_2_Hb_R_^2^	0.997	0.996	0.987	0.973	0.991 (0.005)^†^	0.996	0.990	0.998	0.998	0.955	0.987	0.976	*∗∗*	0.988	0.930	*∗∗∗*	0.913
O_2_Hb_HVT_	15.8	17.5	26.0	20.0	24.6 (6.5)^†^	68.0	51.3	94.5	42.6	223.1	31.1	399.3	*∗∗*	42.5	32.9	*∗∗∗*	48.6

## Data Availability

The analysed data used to support the findings of this study are available from the corresponding author upon request.
